# A Novel Approach for Microencapsulating Salt Hydrate-Based Phase Change Materials

**DOI:** 10.3390/polym17101322

**Published:** 2025-05-13

**Authors:** Jaswinder Sharma, Georgios Polizos, Charl J. Jafta, Siddhant Datta, Kyle R. Gluesenkamp, Kashif Nawaz

**Affiliations:** 1 Oak Ridge National Laboratory, Electrification and Energy Infrastructures Division, Oak Ridge, TN 37831, USA; 2 Building Technologies Research and Integration Center, Oak Ridge National Laboratory, Oak Ridge, TN 37831, USA; 3 Oak Ridge National Laboratory, Surface Chemistry & Catalysis Group, Chemical Sciences Division, Oak Ridge, TN 37831, USA

**Keywords:** phase change materials, salt hydrates, thermal energy storage, electrospinning, core-shell fibers

## Abstract

Energy storage technologies, particularly those utilizing phase change materials (PCMs), have gained attention for their high energy density and efficient thermal management. PCMs, which store energy through solid-liquid phase transitions, can efficiently capture and release thermal energy, but face the challenge of leakage during the phase change process. Inorganic PCMs, such as salt hydrates, offer high energy storage capacity, but are difficult to encapsulate due to their corrosive nature. Conventional encapsulation techniques for inorganic PCMs are limited, particularly for scalable applications. In this work, we present an innovative method for the encapsulation of salt hydrate-based inorganic PCMs (CaCl_2_·6H_2_O) using co-axial electrospinning. The process involves the creation of co-axial fibers, with salt hydrate as the core and polymer (e.g., PVP) as the outer shell, effectively preventing leakage and improving the stability of the PCM. This approach demonstrates the potential for scalable microencapsulation of inorganic PCMs, marking the first report of using co-axial electrospinning for this purpose. This novel technique could contribute to enhancing the performance and applicability of PCMs in thermal energy storage systems and other energy efficiency applications.

## 1. Introduction

The world’s energy demand is increasing every day due to global growth. To meet this demand, the use of traditional energy sources alone is not a sustainable choice due to their limited supply and geopolitical risks [1]. Recently, to diversify the energy sources, the push for other energy sources, such as wind and solar power, has been gaining traction globally. While these energy sources (e.g., solar) can provide sufficient energy for the entire world, the energy obtained from these sources is inconsistent [2,3,4,5]. For example, solar energy (e.g., heat obtained from sunlight) peaks during the day, but there is no solar energy available at night [2,3,4,5]. To address this challenge, technologies are being developed to store solar energy during peak times and make it available when needed [6,7,8,9,10,11]. Among these energy storage technologies, the most common approach is thermal energy storage, using either sensible or latent heat storage methods [12,13,14].

Sensible heat storage is achieved by raising the temperature of a material (e.g., water, sand, etc.) and cooling it when required. This heating and cooling cycle facilitates energy storage and release as needed. In contrast, latent heat storage involves changing the phase of a material (e.g., from solid to liquid) and releasing the energy during a reverse phase change (e.g., from liquid to solid) [13,14,15,16]. While sensible heat storage is easy to use and widely available, it has lower energy storage density, requiring large volumes of material to store the energy [17,18]. On the other hand, latent heat storage materials—known as phase change materials (PCMs)—have a higher energy storage density and can store and release energy without significant temperature fluctuations during the process [19,20].

PCMs are highly sought after for various applications, ranging from enhanced building envelope energy efficiency to battery thermal management systems [21,22,23]. PCMs can be categorized into four types based on the phase transition process: solid–liquid, liquid–gas, solid–gas, and solid–solid [23,24]. Among these, solid-to-liquid (or liquid-to-solid) PCMs are commonly used due to their high energy storage density (∆H_f_ ≈ 100–230 J/g) [23,24]. However, these PCMs face a major technical challenge: leakage [19,20,21,22,23,24,25]. Leakage occurs when the PCM is in liquid form or during the solid-to-liquid phase transition, leading to messy applications and the loss of PCM with each cycle. To address this issue, PCMs are often encapsulated within a secondary material, contained in a secondary container, or filled within a porous material [26,27,28].

PCMs are mainly divided into two types: (1) organic PCMs and (2) inorganic PCMs [21,22,23,24,25]. Organic PCMs (e.g., paraffin) are commonly used because they are easy to encapsulate with a secondary material coating, reducing the risk of leakage. However, organic PCMs are expensive, have lower energy storage efficiency, and are flammable [21,22,23,24,25]. In contrast, inorganic PCMs are inexpensive, have higher energy storage capacity, and are non-flammable [19]. However, their use is challenging due to their corrosive nature and difficulty in encapsulation [19,29,30]. Common techniques used to encapsulate PCMs include sol-gel, in-situ polymerization, complex coacervation, and solvent extraction [31,32,33,34,35,36,37]. While these methods are effective for organic PCMs, inorganic PCMs, being water-soluble, are difficult to encapsulate using conventional techniques. Some progress has been made in microencapsulating inorganic PCMs using the emulsification technique [31,32,33,34,35,36,37], but a widespread, scalable strategy for the microencapsulation of salt hydrates remains lacking.

**Figure 1:**
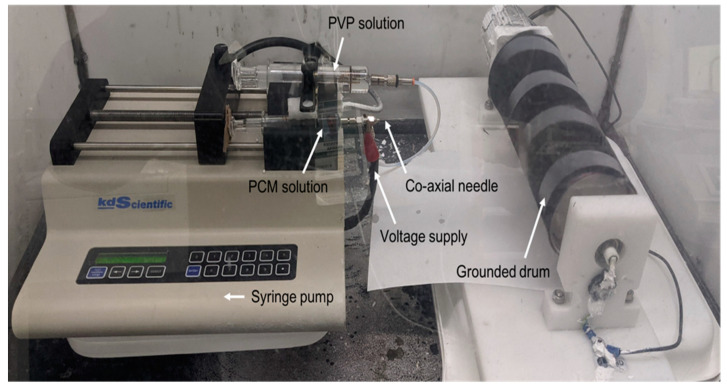
A digital photograph of our setup used for co-axial electrospinning of the salt hydrate PCM core and polymer shell.

In this study, we demonstrate an unconventional technique for encapsulating salt hydrate-based inorganic PCMs (CaCl_2_·6H_2_O). The method involves co-axial electrospinning, where the core is made of the salt hydrate (CaCl_2_·6H_2_O) and the outer shell is made of a polymer. To perform this encapsulation, we prepared two solutions: a salt hydrate solution and a polymer solution. The salt hydrate solution was placed in one syringe, and the polymer solution in another. Both syringes were connected to a co-axial needle, with the salt hydrate solution connected to the inner needle and the polymer solution to the outer needle. The co-axial needle was then connected to a high-voltage supply. When the syringes were pushed by a syringe pump and voltage was applied, concentric solution droplets emerged from the needle tip, forming co-axial fibers (with the salt hydrate core and polymer shell, e.g., PVP). The fibers were collected on a rotating drum or other surface. A digital photograph of our electrospinning setup is shown in Figure 1. Although co-axial electrospinning has been used for the microencapsulation of organic PCMs [38,39,40,41], this is the first report to demonstrate its use for the microencapsulation of inorganic PCMs.

## 2. Materials and Methods

This is an open access article, free of all copyright, and may be freely reproduced, distributed, transmitted, modified, built upon, or otherwise used by anyone for any lawful purpose. The work is made available under the Creative Commons CC0 public domain dedication.

### 2.1. Chemicals

CaCl_2_ was purchased from Sigma Aldrich, and CaCl_2_·6H_2_O was prepared by adding a stoichiometric amount of water to the CaCl_2_. Polymers (Polyvinyllpyrrolidone; PVP; MW-1,300,000 and Poly (methyl methacrylate); PMMA; MW-960,000) were purchased from Sigma Aldrich. Dimethylformamide (DMF) was purchased from Fisher Chemicals. The co-axial needle (100-10-COAXIAL-1814) was purchased from Ramé-Hart Instrument Co., Succasunna, NJ, USA.

CaCl_2_·6H_2_O (salt hydrate) solution was prepared by adding an appropriate amount of water into anhydrous CaCl_2_, followed by sonication in a water bath heated at 35 °C for 5–10 min. Similarly, PVP and PMMA polymer solutions were prepared by adding 1.0 g of polymer to 9.0 g of DMF, followed by stirring using a magnetic stir bar (Fisher Scientific, Hampton, NH, USA) at 50 °C overnight. Overnight stirring is recommended to get a nice homogeneous solution.

### 2.2. Electrospinning

An in-house-made electrospinning setup was used for encapsulating the PCM (Figure 1). A syringe pump (Kd Scientific Kds-200 Dual Syringe Pump) with slots for two syringes was used. One glass syringe containing the salt hydrate solution was directly connected to the co-axial needle while the second, containing a polymer solution, was connected to the needle through a PTFE tube (100-10-PTFE-KIT from Ramé-Hart Instrument Co.). Voltage (14 kV) was applied to the needle tip by using an alligator clip. A grounded drum collector was used for collecting fibers. A flow rate of 1 mL/h and a needle tip to drum collector distance of ≈16 cm were used.

### 2.3. Scanning Electron Microscope (SEM) Analysis

Scanning Electron Microscopy (SEM) imaging was performed using a Merlin 200 microscope at the Center for Nanophase Materials Sciences, a DOE user facility at Oak Ridge National Lab. To minimize the charging on the fiber surface, fibers were deposited on a silicon wafer, which was in turn attached to the SEM stub using a carbon tape, before performing the SEM imaging.

### 2.4. X-Ray Photoelectron Spectroscopy Analysis

X-ray photoelectron spectroscopy (XPS) was performed using a Thermo Scientific (Waltham, MA, USA) Model K-Alpha XPS instrument. The instrument utilizes monochromated, micro-focused, Al Kα X-rays (1486.6 eV) with a variable spot size (i.e., 30–400 µm). Analyses of the sample were performed with the 400 µm X-ray spot size for maximum signal and to obtain an average surface composition over the largest possible area. The instrument has a hemispherical electron energy analyzer equipped with a 128-channel detector system. Base pressure in the analysis chamber is typically 2 × 10^−9^ mbar or lower. The fiber sample was mounted on double-sided tape fixed to a clean glass slide. After transferring the sample into the analysis chamber, a survey spectrum (pass energy = 200 eV) was acquired to determine all elements present. Next, high-resolution core-level spectra (pass energy = 50 eV) were acquired for detailed chemical state analysis. All spectra were acquired with the charge neutralization flood gun (combination of low-energy electrons and argon ions) turned on to maintain a stable analysis condition. The typical pressure in the analysis chamber with the flood gun operating is 2 × 10^−7^ mbar. Data were collected and processed using the Thermo Scientific Avantage XPS software package (v.5.96).

### 2.5. Thermogravimetric Analysis

Thermogravimetric Analysis (TGA) was performed in an air atmosphere on a TA Instruments TGA-2950 from ambient temperature to 800 °C at a heating rate of 10 °C min^−1^.

## 3. Results and Discussion

Electrospinning is a widely used technique for producing nanofibers and microfibers by applying a high-voltage electric field to a polymer solution or melt. The process begins with the polymer solution being drawn from a syringe through a needle, where the electric field causes the solution to form a fine jet. As the jet travels toward a grounded collector, the solvent evaporates, and the polymer solidifies into fibers that are collected as nonwoven mats or aligned structures. Electrospinning offers several advantages, including the ability to produce fibers with extremely small diameters, high surface area-to-volume ratios, and versatile material choices.

**Figure 2:**
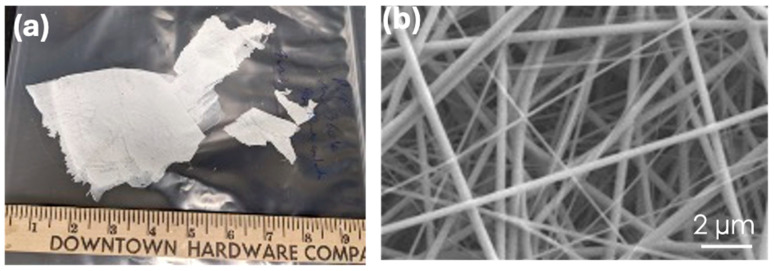
Photo of as-spun PVP fibers (**a**) and SEM image of the same fibers (**b**).

It is known that only materials with fibrous chains or long one-dimensional structures can be electrospun, while materials with a granular composition cannot. General materials that can be electrospun include polymers, carbon nanotubes, and other substances composed of long chains or fibrous structures. Initial efforts to optimize the conditions are summarized in Table 1. Briefly, salt hydrate (CaCl_2_·6H_2_O) alone cannot be electrospun; it always requires an additional material, such as a polymer, to enable electrospinning. In contrast, as expected, the shell material can be electrospun alone. Figure 2 shows the photo of the nonwoven mat and the SEM image of PVP fibers produced by electrospinning.

**Figure 3:**
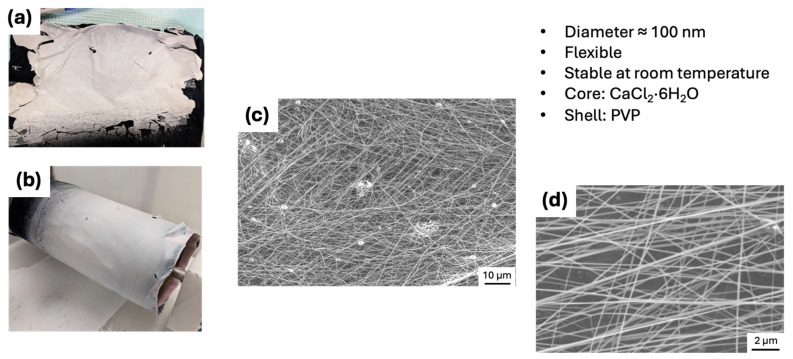
CaCl_2_·6H_2_O (core)-PVP (shell) fibers. As-spun fiber mat on a piece of cloth (**a**), as-spun fiber mat on the collector drum (**b**), low-magnification SEM image of fibers (**c**), and (**d**) high-magnification SEM image of fibers (**d**).

Electrospinning of core-shell fibers was started by a low concentration of CaCl_2_·6H_2_O (core) solution in water. Ultimately, we successfully electrospun core-shell fibers using 97 wt.% CaCl_2_·6H_2_O in water (core) and 10 wt.% PVP in DMF (shell) solutions. During the electrospinning process, the solvent evaporates from the PVP shell, leaving behind a solid PVP polymer shell. Similarly, a small amount of water also evaporates from the CaCl_2_·6H_2_O core; however, the addition of 3–4% extra water in the core solution compensates for this loss while retaining a good quantity of water molecules inside the CaCl_2_·6H_2_O core. Figure 3a shows the as-spun core-shell fibers on a piece of cloth, and Figure 3b shows the electrospun fibers on the same piece of cloth still wrapped around the drum collector. Figure 3c,d show SEM images of the same fibers, revealing that the fibers have a diameter of approximately 100 nm. The fiber diameter can be adjusted by altering the electrospinning conditions. For example, increasing the voltage and the distance between the drum collector and the needle tip results in smaller diameter fibers [42].

As shown in Figure 2, the as-spun core-shell fibers form a uniform mat on the cloth wrapped around the rolling drum. The cloth was wrapped around the drum to prevent drum contamination and facilitate the removal of the fiber mat.

To estimate the yield of electrospinning, we pre-weighed the piece of cloth before electrospinning fibers onto it. A measured amount of salt hydrate and polymer was then electrospun onto the cloth using co-axial electrospinning. By subtracting the weight of the bare cloth from the weight of the cloth with fibers, we calculated a yield of ≥75%. The remaining 25% of the material was lost during electrospinning, as sometimes the co-axial stream broke, causing the material to fall onto the ground instead of wrapping around the cloth. The detailed weights of the different materials are provided in Table 2.

**Figure 4:**
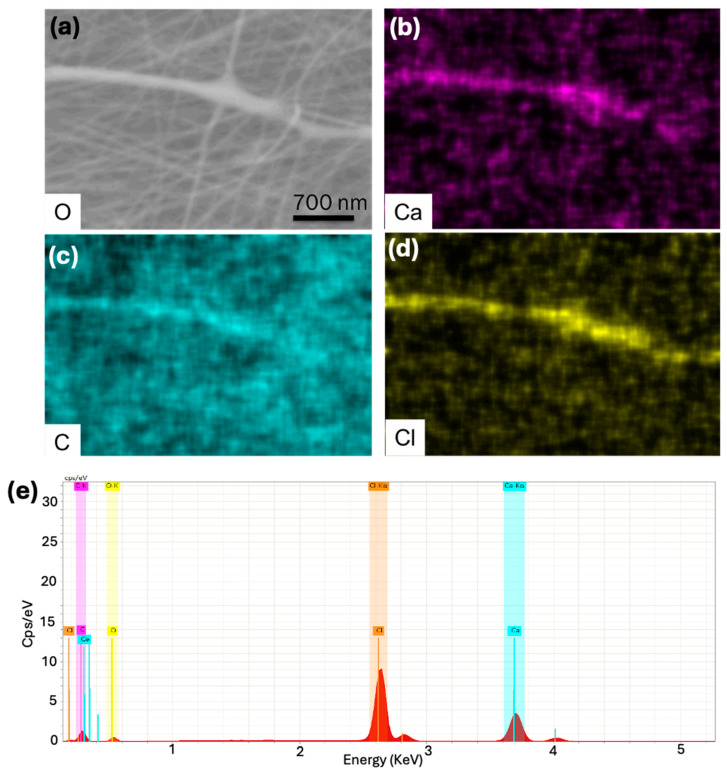
EDAX analysis of as-spun core-shell fibers. (**a**) SEM image of core-shell fibers, (**b**–**d**) EDAX maps of core-shell fibers, and (**e**) EDAX spectrum of core-shell fibers.

To confirm that the electrospun fibers are not composed of polymer alone and contain salt hydrate, we performed EDAX analysis on the core-shell fibers. The EDAX analysis (Figure 4a,b) clearly showed the presence of ‘Ca’ and ‘Cl’ elements in the fibers. It is important to note that the shell polymer (PVP) contains only ‘C’, ‘O’, and ‘N’; thus, the presence of ‘Ca’ and ‘Cl’ confirms that CaCl_2_·6H_2_O is encapsulated inside the polymer shell. Additionally, it is worth noting that EDAX is not a surface technique and can detect elements up to a depth of approximately 2 µm.

**Figure 5:**
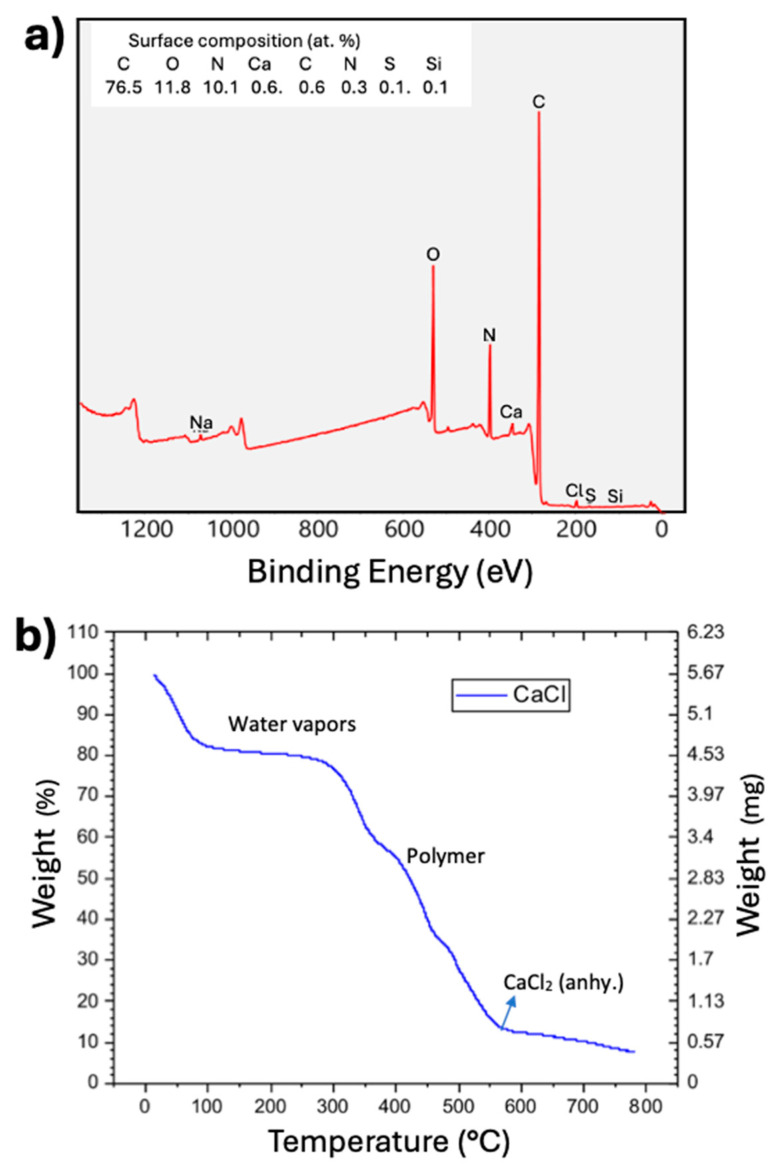
XPS analysis (**a**) and TGA analysis (**b**) of the CaCl_2_·6H_2_O (core)-PVP polymer (shell) fibers.

To confirm the core-shell nature of the fibers, X-ray photoelectron spectroscopy (XPS) was performed (Figure 5a). XPS is a surface analysis technique with a detection depth in the range of 5–10 nm. Only 0.6 at% of ‘Ca’ and 0.6 at% of ‘Cl’ were detected on the surface of the fibers. If CaCl_2_ had penetrated through the polymer coating, the Ca:Cl ratio should be 1:2. However, the observed 1:1 ratio suggests that these elements may have resulted from contamination during sample preparation. Therefore, it is inferred that minimal leakage of CaCl_2_·6H_2_O occurred through the fiber shell. The absence of CaCl_2_ on the fiber surface rules out the possibility that the fibers are composites of CaCl_2_·6H_2_O and polymer. The presence of CaCl_2_ in the EDAX maps and its minimal presence in the XPS analysis further confirms the (1) core-shell nature of the fibers and (2) minimal leakage of CaCl_2_ through the PVP shell.

The TGA plot (Figure 5b) shows that up to approximately 300 °C, water molecules and other contaminants evaporate from the fiber surface and core. The polymer begins to disintegrate at about 300 °C and completely burns out at around 500 °C. The 12.5 wt.% residue remaining after 500 °C is CaCl_2_. When CaCl_2_ is converted to CaCl_2_·6H_2_O by adding six water molecules, it indicates that the core-shell fibers contain approximately 25 wt.% CaCl_2_·6H_2_O. We are currently working to increase the CaCl_2_·6H_2_O loading capacity of the electrospun core-shell fibers from 25% to over 90%. The increased loading capacity will enhance energy storage and, in turn, improve both gravimetric and volumetric thermal energy storage density.

**Figure 6:**
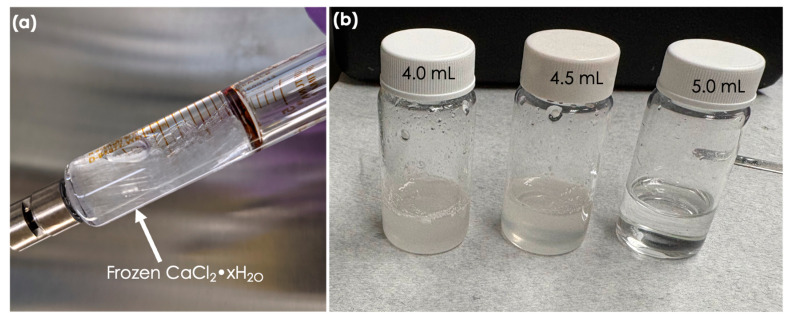
(**a**) Digital photograph of frozen PCM in the syringe, (**b**) digital photographs showing the required amount of water for making the clear solution for 5 g of anhydrous CaCl_2_.

During the co-axial electrospinning process, we observed that when using a solution of 97–100% CaCl_2_·6H_2_O at a lab temperature of approximately 22 °C, the CaCl_2_·6H_2_O solution freezes in the syringe (Figure 6a). Freezing the CaCl_2_·6H_2_O solution results in clogging the needle and syringe, which causes back pressure on the syringe pump. We found that when CaCl_2_·6H_2_O freezes in the syringe and the syringe pump continues to push, the pump either becomes stuck and stops pushing, or in some cases, the pump breaks the glass syringe. Therefore, care must be taken to minimize the freezing of CaCl_2_·6H_2_O and avoid damaging the syringe or syringe pump.

To prevent freezing, either the electrospinning temperature should be maintained above 28 °C, or the CaCl_2_·6H_2_O solution should be diluted with water (e.g., using 90–95% CaCl_2_·6H_2_O instead of 97–100%). To make CaCl_2_·6H_2_O from anhydrous CaCl_2_, 4.86 mL of water is required for every 5.0 g of CaCl_2_. Therefore, to prevent freezing, it is recommended to use an excess of water, around 5.00–5.25 mL of water for every 5.0 g of CaCl_2_. Additionally, it was observed that if the water amount was less than 4.86 mL for 5.0 g of anhydrous CaCl_2_, the obtained solution was turbid, and a clear solution was only obtained when the water was equal to or more than 4.86 mL. Figure 6b shows the digital photographs of solutions obtained after adding 4 mL, 4.5 mL, and 5.0 mL of water to 5.0 g of anhydrous CaCl_2_. Solutions were prepared by mixing the CaCl_2_ through sonication in a water bath at 35 °C.

**Figure 7:**
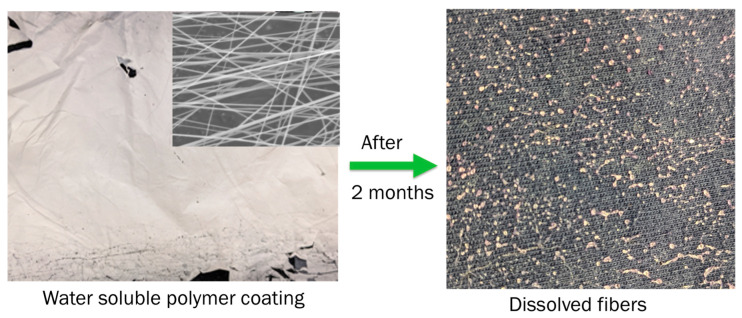
Dissolution of encapsulated fibers. Fiber mat on cloth and the SEM image of the fibers in the inset (**left**), the dissolved fiber mat in the absorbed water (**right**).

Although the electrospinning of core-shell fibers with a CaCl_2_·6H_2_O core and PVP shell was successful, we observed that PVP, being water soluble and highly permeable to water, allows water vapors to pass through it. The CaCl_2_·6H_2_O inside the fibers absorbs excess water. Over a period of two months, this excess absorbed water caused the fiber mat to dissolve (Figure 7). Therefore, while water-soluble polymers are easy to use in co-axial electrospinning, we found that the durability of the encapsulated fibers is not very high. As a result, these fibers are not suitable for long-duration energy storage applications.

**Figure 8:**
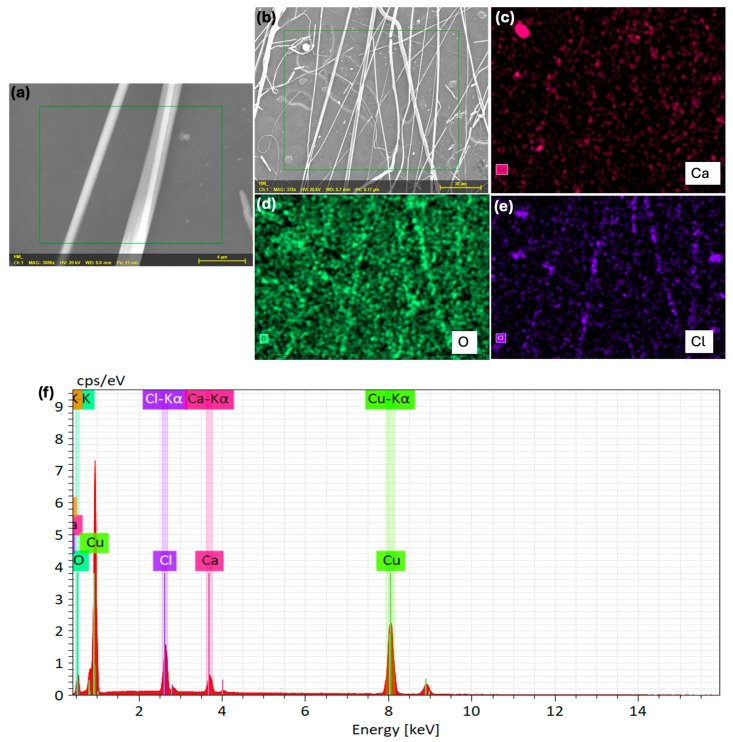
SEM and EDAX analysis of core-shell fibers made of CaCl_2_·6H_2_O core and PMMA shell. (**a**) Zoomed in SEM image of the fibers, (**b**) SEM image of the fibers, (**c**–**e**) EDAX maps of the same fibers, and (**f**) EDX spectrum of the same fibers.

To address the issue of fiber shell dissolution in absorbed water, we replaced the hydrophilic polymer (PVP) with the hydrophobic polymer polymethyl methacrylate (PMMA). A 97 wt.% CaCl_2_·6H_2_O solution in water was used as the core solution, and a 10 wt.% PMMA solution in DMF was used as the shell solution. Figure 8 shows the SEM image, EDAX maps, and EDAX spectrum of these core-shell fibers. The EDAX maps and spectrum clearly revealed the presence of ‘Ca’ and ‘Cl’, confirming the core-shell nature of these fibers.

Compared to the core-shell fibers with PVP as the shell, the fibers with PMMA as the shell exhibited weaker signals for ‘Ca’ and ‘Cl’. This suggests that either the percentage of CaCl_2_ encapsulated is lower when PMMA (a hydrophobic polymer) is used as the shell polymer, or the shell thickness is greater, leading to a larger overall fiber diameter. An increased shell thickness could result in a weaker signal for ‘Ca’ and ‘Cl’, as both elements are located in the core. As expected, the fiber diameter was quite large, approximately 1.0–3.0 µm, indicating that the corresponding shell thickness was also greater, resulting in a weaker EDAX signal.

Since the core solution (CaCl_2_·6H_2_O) and shell solution were immiscible with each other, the quality of the electrospun fibers was not as high as when the core and shell solutions were miscible, such as when PVP was used as the shell solution. The main challenge when electrospinning immiscible solutions is the co-agglutination at the syringe tip, which leads to clogging of the needle. Therefore, the clogged material at the syringe tip had to be removed intermittently. Further optimization is needed to select a shell polymer that is compatible with the core solution and enables smooth electrospinning of fibers.

**Figure 9:**
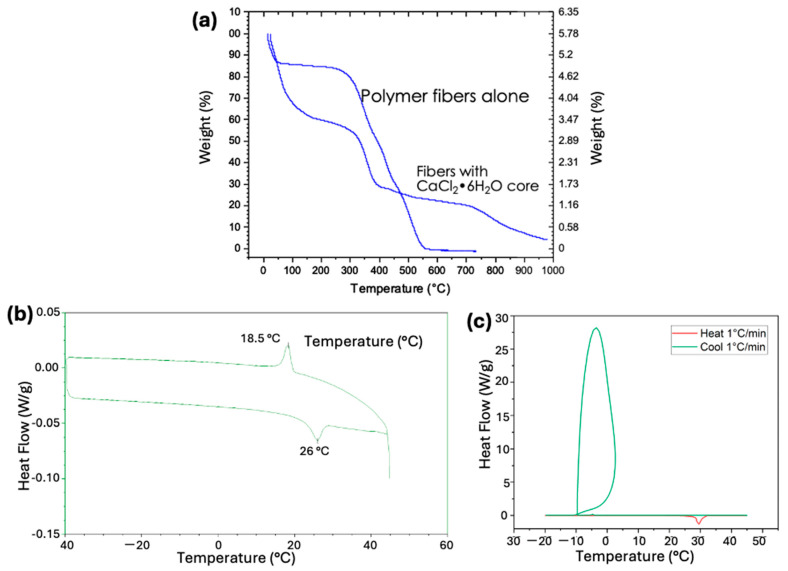
(**a**) TGA analysis of electrospun fibers made of PVP core and PMMA shell, and of CaCl_2_·6H_2_O core and PMMA shell; (**b**) DSC thermograms of the same fibers, and (**c**) DSC thermogram of the unencapsulated CaCl_2_·6H_2_O.

TGA analysis of PVP core and PMMA shell (core and shell are both made of polymer) shows that at 550 °C, all the polymers burn out, leaving almost nothing behind (Figure 9a). However, TGA analysis of the CaCl_2_·6H_2_O (core) and PMMA shell shows that at approximately 550 °C, about 25% of the residue remains. This residue is CaCl_2_, and when we convert CaCl_2_ to CaCl_2_·6H_2_O by adding six water molecules, it indicates that the core-shell fibers contain approximately 50 wt.% CaCl_2_·6H_2_O. The TGA plots show that up to approximately 300 °C, water molecules and other contaminants evaporate from the fiber surface and core. The polymer begins to disintegrate around 300 °C and completely burns out by approximately 500 °C. The residue remaining after 500 °C is CaCl_2_. TGA analysis showed that fibers with a PMMA shell have a higher loading (50 wt.%) of CaCl_2_·6H_2_O compared to those with a PVP shell (25 wt.%). This increased loading may be due to the larger diameter (1–3 µm) of the fibers with the PMMA shell, compared to the smaller diameter (≈100 nm) of the fibers with the PVP shell.

DSC analysis (Figure 9b) of the fibers showed two peaks: one at 18.5 °C, indicating the freezing of CaCl_2_·6H_2_O, and another at 26 °C, indicating the melting of CaCl_2_·6H_2_O. DSC analysis indicates that the phase change material is active and participates in thermal cycling. Furthermore, DSC analysis reveals that the freezing (18.5 °C) and melting (26 °C) points of encapsulated CaCl_2_·6H_2_O are lower than the theoretical freezing (≈30 °C) and melting points (30 °C) of pure CaCl_2_·6H_2_O. Though the theoretical freezing point is higher, our DSC studies on pure CaCl_2_·6H_2_O showed severe supercooling resulting in a very low freezing point (−10 °C) while showing a melting point equal to the theoretical value (30 °C) (Figure 9c). Additionally, DSC experiments (Figure 9b,c) showed that the latent heat of fusion was only ≈39 J/g instead of ≈170 J/g, as expected for the pure unencapsulated CaCl_2_·6H_2_O. This low value of latent heat may be due to (1) lower loading (≈50%) of CaCl_2_·6H_2_O, as shown by the TGA results, and (2) incomplete participation of salt hydrate in thermal cycling.

## 4. Conclusions

CaCl_2_·6H_2_O salt-hydrate phase change material (PCM) has been successfully encapsulated using the co-axial electrospinning technique. Electrospinning of salt hydrates alone is not possible; however, they can be electrospun by mixing them with a polymer or by having a polymer shell. Clogging the nozzle opening is a common issue due to the immiscible nature of the polymers or solvents. Clogging can also occur from the freezing of the salt hydrate under electrospinning temperature conditions. Water-soluble coating polymers are not suitable for the long-term encapsulation of salt hydrates, as their porous nature allows salt hydrates to absorb water molecules from the outside through the polymer shell. Over time, the polymer shell dissolves in the absorbed water, causing the fibers to transform into droplets containing dissolved CaCl_2_·6H_2_O and polymer. However, hydrophobic polymer coatings provides longer-term encapsulation for the salt hydrates. We anticipate that this work will pave a path for the encapsulation of inorganic phase change materials by using electrospinning or similar core-shell formation techniques.

## Figures and Tables

**Table 1:** A summary of material compositions with the capability or incapability to form electrospun fibers.

Composition	Electrospinning
CaCl_2_·6H_2_O alone	No
PVP alone	Yes
90% CaCl_2_·6H_2_O in water (core) + 10% PVP in DMF (shell)	Yes

**Table 2:** Calculation of the yield of the electrospinning process.

Entity Name	Weights
CaCl_2_·6H_2_O	1.0 g
PVP	0.1 g
Piece of cloth without fibers	9.13 g
Piece of cloth with fibers	9.96 g
Amount of fibers	0.75 g
Electrospinning yield	≈75%

## Data Availability

The data presented in this study are available on request from the corresponding author.
